# What Type of Food Can Older Adults Masticate?: Evaluation of Mastication Performance Using Color-Changeable Chewing Gum

**DOI:** 10.1007/s00455-017-9807-1

**Published:** 2017-05-04

**Authors:** Shinichi Wada, Nobuyuki Kawate, Masazumi Mizuma

**Affiliations:** 1 Department of Rehabilitation Medicine, Moriyama Rehabilitation Clinic, 1-11-17 Nishi-Nakanobu, Shinagawa-ku, Tokyo, 142-0054 Japan; 2 Department of Rehabilitation Medicine, Showa University School of Medicine, 1-5-8 Hatanodai, Shinagawa-ku, Tokyo, Japan

**Keywords:** Deglutition, Masticatory performance, Food bolus, Color-changeable chewing gum

## Abstract

This study determines if older adults can masticate regular foods via a simple test conducted using a color-changeable chewing gum. Seventy-nine consecutive inpatients of our clinic receiving rehabilitation and general medicine were assessed for eligibility. The inclusion criterion was >65 years. Thirty patients consented to participate. The main outcome variable was the food bolus texture at the swallowing threshold for five regular foods. The main explanatory variable was the a* value of the color-changeable chewing gum after 120 s of chewing (a* represents the degree of color between red and green, and a positive a* value indicates red). The mean age ± standard deviation of the participants was 81.6 ± 8.6 years, and 40% were men. Participants being able to prepare the food with suitable texture for swallowing was positively associated with the a* values in boiled rice, ginger-fried pork loin, boiled fish-paste, and rice cracker (Crude OR 1.18, 1.15, 1.17, and 1.50; *P* < 0.001, = 0.026, <0.001, and <0.001, respectively). The cut-off a* values had markedly high specificities (1.0) for boiled rice and boiled fish-paste and high sensitivities (0.86–0.94) for three foods, except boiled rice. We believe that mastication evaluation using the color-changeable chewing gum is not only useful but also extremely practical, even for older adults in a wide range of settings, including an individual’s home. This approach would lead to a reduction in unnecessary mechanically altered or pureed food for older adults who can eat pureed food and safely provide palatable food.

## Introduction

Although patients are sometimes unaware of the disorder, oropharyngeal dysphagia is a highly prevalent clinical condition as it affects 10–30% of adults aged 65 and above [1] and more than 51% of institutionalized elderly patients [2]. Conservative estimates suggest that 8% of the worldwide population experiences difficulty in consuming regular food and fluids because of dysphagia [3]. Having fewer teeth inhibits masticatory ability, which disturbs smooth swallowing [4].

The process of chewing and swallowing food is very complex. The skill required to break down solid food, mix it with saliva, collect it into a cohesive bolus, and transport it to the posterior of the oral cavity for swallowing can be challenging to nearly impossible for individuals with oropharyngeal dysphagia. General clinicians and caregivers usually do not evaluate all these skills; as a result, unnecessary mechanically altered or pureed food might be provided for elderly patients, which might have a negative impact on their quality of life [5]. Therefore, we need to more comprehensively, simply, and practically determine the types of food a person can eat.

In previous studies, mastication performance has often been evaluated by measurement of an individual’s ability to grind or pulverize a test food by chewing. The degree of food breakdown is then determined by sieving [6, 7]; however, it is not considered a simple and practical method. Another popular method evaluates an individual’s ability to mix and knead a food bolus. Also, a two-color chewing gum [8, 9] and paraffin wax [10, 11] have been used as test items to quantify mixing ability.

The method using a color-changeable chewing gum demonstrates mastication performance based on mixing ability as well as the comminuting ability determined by the sieve test [12]. Changes in the color values of a color-changeable chewing gum reliably reflected mastication performance of individuals with natural dentition and those with complete dentures [13]. A color scale was developed for visual assessment of the gum to evaluate mastication performance [14].

Although these useful and practical scales require only a few minutes from the user to reach an objective evaluation, this evaluation cannot determine the types of food a person can eat. If we could determine the food type a person can masticate, through a simple test using a color-changeable chewing gum, then it would be useful not only in laboratories and specialized institutions but also in a wider range of settings, such as an individual’s home.

This study aims to evaluate whether older adults can masticate regular foods via a simple test conducted using a color-changeable chewing gum. This approach may lead to reductions in unnecessary mechanically altered or pureed food for an older adult who can eat pureed food and to safely provide palatable food.

We designed this study to assess the hypothesis that good mastication ability, evaluated using a color-changeable chewing gum, is associated with a suitable food bolus texture at the swallowing threshold.

## Methods

### Setting

The Moriyama Rehabilitation Clinic, located in a residential area in a special ward in Tokyo Metropolis, has 19 beds for inpatients and provides rehabilitation medicine and general medicine for inpatients and outpatients to the local community.

### Subjects

All inpatients of our clinic were assessed for eligibility. The inclusion criterion was ≥65 years of age. Exclusion criteria included the inability to consume adequate food by oral ingestion, such as severe dysphasia, the inability to follow instructions, such as chewing and spitting out the gum, medical instability or other medical conditions that could confound the results, and patient refusal to participate. All participants or their representatives provided written informed consent.

## Materials

### Color-Changeable Chewing Gum and Color Measurement

**Fig. 1:**
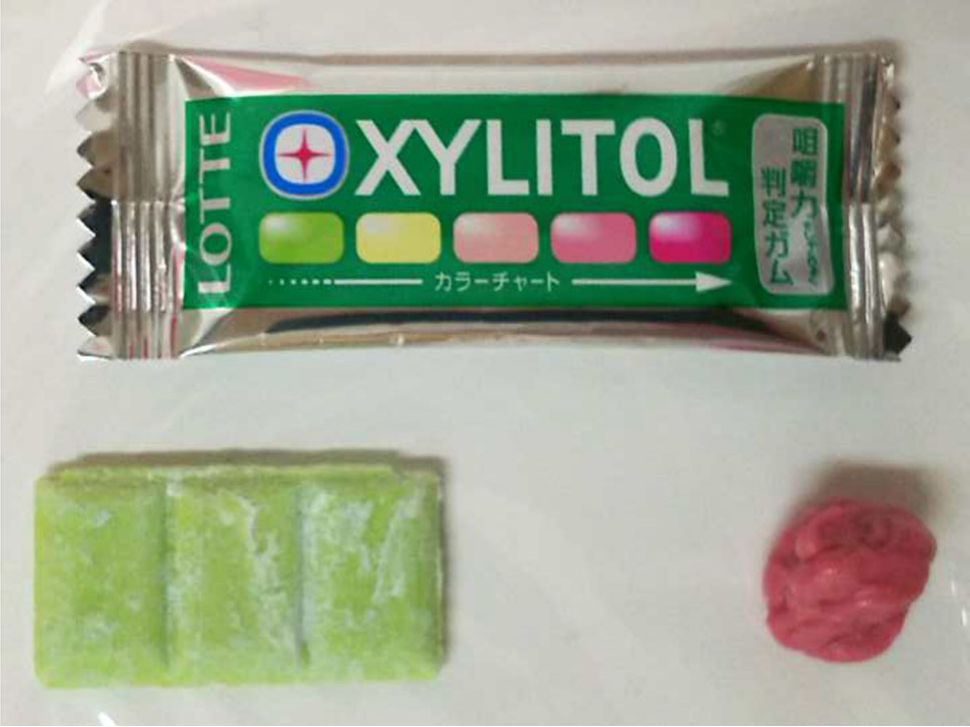
The color-changeable chewing gum (70 × 20 × 1 mm^3^; 3.0 g). The gum changes color from *yellowish-green* (*right side*) to *red* (*left side*) after chewing. The *red* dye is pH-sensitive and changes color under neutral or alkaline conditions. Citric acid maintains a low internal pH of the *yellowish*-*green* gum before chewing. As chewing progresses, the gum changes to *red* because the *yellow* and *blue* dyes seep into the saliva and the *red* color appears as a result of citric acid elution. Changes in the color values of the gum reflect the comprehensive ability of mastication

The color of the chewing gum (70 × 20 × 1 mm^3^; 3.0 g; Masticatory Performance Evaluating Gum XYLITOL, Lotte, Tokyo, Japan; Fig. 1) changes from yellowish-green to red when chewed. The gum base contains xylitol, citric acid, and red, yellow, and blue dyes, and it can be adjusted to not adhere to denture materials, which allow easy chewing, even by complete denture wearers who have reduced occlusal force. The red dye is pH-sensitive and changes color under neutral or alkaline conditions. Citric acid maintains a low internal pH of the yellowish-green gum before chewing. As chewing progresses, the gum color changes to red because the yellow and blue dyes seep into the saliva and the red color appears as a result of citric acid elution. Changes in the color values of the gum reflect the comprehensive abilities of mastication, such as dental occlusion, salivary secretion, and tongue movements during chewing [12, 13].

Color was measured immediately after chewing for 120 s. The chewed gum was flattened to a thickness of 1.5 mm in polyvinylidene chloride films by compression between two glass plates and measured at five points using a colorimeter (CR-13; Konica-Minolta Sensing, Tokyo, Japan) positioned at the center and approximately 5 mm above, below, right, and left of center. Changes in color were visualized as three-dimensional coordinates organized along the L*, a*, and b* axes and evaluated using the CIELAB color system defined by the International Commission on Illumination, where a* represents the degree of color between red and green. Positive values for a* indicate red. The mean values of five points for each of a* were determined.

### Food Samples

We selected five foods mainly from the 35 foods listed in a questionnaire for evaluating mastication function [15]. The foods on the list were classified into five levels according to the degree of mastication difficulty. Foods in the lower level of the list were easier to masticate. In this study, we selected one food from the three middle levels of the list (boiled fish-paste: level two, rice cracker: level three, and fried pork: level four) and our staple foods (boiled rice and sliced white bread). The specific foods in our study were boiled rice (Sato no Gohan; a pack of 200 g prepared rice; the type of rice was Koshihikari, Sato Foods Industries, Niigata, Japan), sliced white bread (Pasco Chojuku; 20 mm in thickness, Shikishima Baking, Aichi, Japan), ginger-fried pork loin (Seven Premium, Seven & i Holdings, Tokyo, Japan), boiled fish-paste (Yuzuki mini red 95 g, Yuzuki, Fukushima, Japan), and rice cracker (Teshioya flavored with stock and salt, Kameda Seika, Niigata, Japan).

### Measurements of Food Bolus Texture at the Swallowing Threshold

Participants were instructed to masticate a mouthful of food in their usual manner and spit out the food bolus immediately before swallow onset. The test foods were administered in the order of boiled rice, sliced white bread, ginger-fried pork loin, boiled fish-paste, and rice cracker. The weight of a mouthful of food before mastication, mastication frequency, mastication time, and the texture of the food bolus spewed were measured. The food texture properties of hardness, cohesiveness, and adherence were measured by the texture profile analysis method determined by the Japanese Ministry of Health, Labour and Welfare. We used a texture analysis instrument (The TA.XT plus Texture Analyser, Stable Micro Systems, Surrey, UK) equipped with a cylindrical plunger (ø 20 mm), a cylindrical cup (int. ø 40 mm), and a 50-N load cell. The bolus underwent two successive compression cycles performed at a constant displacement rate of 10 mm/s. A compression ratio of 67% deformation (the bolus was filled 15 mm in height and the clearance was 5 mm) was chosen. The hardness was determined by the first peak height in the load–time curve, and the adhesiveness was determined by the negative force area for the first bite representing the work required to pull the plunger away from the sample. The cohesiveness was determined by the ratio of the positive force area of the second compression to that of the first compression [16, 17].

Japanese dysphagia diet level two is defined as a rough-surface jelly food with protein, and it is equivalent to the dysphagia pureed designation of the National Dysphagia Diet in the USA [3] as well as level three in the texture-modified food pyramid of Japan. The lower levels in the texture-modified food pyramid are easier to swallow. Levels zero to three in the texture-modified food pyramid are defined as a texture that satisfies three criteria: (1) under 15,000 N/m^2^ in hardness, (2) under 1000 J/m^2^ in adhesiveness, and (3) between 0.2 and 0.9 in cohesiveness. If the food bolus texture was within the zero to three levels, we regarded it as suitable for swallowing.

### Other Measurements

Occlusal contact status was recorded by the Eichner index [18], where class A has a maximum of four supporting zones (minimum of one tooth contact between the antagonist jaws in the premolar and molar region on each side), class B has one to three supporting zones, and class C has no supporting zones. The Eichner index was recorded for variations in supporting zones with and without removable prostheses.

We also performed the repetitive saliva swallowing test, the water swallowing test (3-mL-modified) [19], and the Mann assessment of swallowing ability (MASA) test [20] as only reference.

### Design

This was a cross-sectional study. The main outcome variable was the food bolus texture at the swallowing threshold, which was represented as suitable mastication for swallowing (level zero to three) or poor mastication (above level three) based on the texture-modified food pyramid. The main explanatory variable was the a* value of the color-changeable chewing gum after 120 s of chewing. Our hypothesis was that a high a* value is associated with a suitable food bolus texture at the swallowing threshold.

### Sample Size

We assumed that the mean a* value after 120 s of chewing was 25 in participants who had suitable mastication abilities and 12 in those with poor mastication abilities, and that the standard deviation (SD) of the a* values was 10 (in a previous study, the SD of a* values was approximately five in dentate people) [12]. The level of significance was set at *P* < 0.05, and the desired statistical power of the trial was set at 0.8. We considered that the expected effect size was at least one. With the sample sizes of both groups equal, the required sample size was 17. We assumed unequal sizes and determined that the required sample size for this study was 30.

### Statistical Analysis

Univariate logistic regression analyses were performed to calculate odds ratios (ORs) and 95% confidence intervals and to examine the relationship between suitable preparations for swallowing and each explanatory variable. After the univariate analyses, the a* values (main explanatory variable), age (spontaneous, because it is known to be associated with mastication abilities), and the significant variables were tested in a multivariate logistic regression analysis. We set the cut-off as the a* values that were nearest to the point (sensitivity: 1, 1-specificity: 0) on the receiver–operating characteristic curves in order to increase both sensitivity and specificity.

JMP version 9.0.2 (SAS institute Inc., Cary, NC) was used for all analyses; *P* < 0.05 was considered statistically significant.

## Results

### Participants

Seventy-nine consecutive inpatients of our clinic were assessed for eligibility from December 2014 to August 2015, 12 of which were younger than 65 years. Among the 67 patients, we excluded four patients who had tube feeding or total parenteral nutrition and 17 patients who were medically unstable or not able to follow our instructions because of dementia or aphasia. Of the remaining 46 eligible patients, 30 were willing to participate.

### Characteristics

Table 1 shows the demographic and clinical characteristics of the participants. The mean age ± SD of the participants was 81.6 ± 8.6 years, and 12 participants (40%) were men. Rehabilitation for bone and joint disease or cerebrovascular disease accounted for 80% of the admissions. The Functional Independence Measure indicated that the average participant’s motor function was moderate assistance or minimal contact assistance, but their average cognitive function was modified independence.

**Table 1:** Participant characteristics

Characteristic	Values are mean ± SD (median) or *n* (%)
Age (years)	81.6 ± 8.6
Sex (male)	12 (40%)
Reason for admission: rehabilitation for cerebrovascular disease	9 (30%)
Reason for admission: rehabilitation for bone and joint disease	15 (50%)
Functional independence measure (FIM) at admission (median)	77.5 ± 26.6 (80.5)
The motor subscale of FIM at admission (median)	49.7 ± 20.7 (55)
The cognition subscale of FIM at admission (median)	27.8 ± 7.8 (31.5)
Repetitive saliva swallowing test	2.8 ± 1.6 (3)
MASA^a^	190.5 ± 9.6 (194.5)
The a* values of the color-changeable chewing gum after 120 s of chewing	21.6 ± 11.6 (median: 27.8, maximum: 32.8, minimum: −9.9)
Mastication frequency of the gum over 120 s	150 ± 34 (median: 156, maximum: 214, minimum: 40)
The water swallowing test (3 mL modified)^b^	
#5	27 (90%)
#4	1 (3%)
#3	2 (7%)
Eichner index (supporting zones without removable prostheses)^c^	
A	10 (33%)
B	7 (23%)
C	13 (43%)
Eichner index (supporting zones with removable prostheses)^c^	
A	23 (77%)
B	6 (20%)
C	1 (3%)

^a^Total score of The Mann Assessment of Swallowing Ability

^b^Criteria #3: Swallowed 3 mL of cold water successfully, but with choking and/or wet hoarseness. Criteria #4: Swallowed 3 mL of cold water successfully without choking or wet hoarseness. Criteria #5: Criteria #4, plus, 2 successful swallowing within 30 s

^c^Class A: maximum of four supporting zones (minimum of one tooth contact between the antagonist jaws in the premolar and molar region on each side). Class B: one to three supporting zones. Class C: no supporting zone

Without removable prostheses, more participants were classified as Eichner class C, which has no supporting zone, than any other class. However, including occlusal supporting zones of the removable dentures, the percentages were 77% in Eichner class A, which has a maximum of four supporting zones, and 3% in class C. The mean total score ± SD of MASA was 190.5 ± 9.6. Dysphagia is unlikely if the total score of MASA is more than 178 [20].

### Food Bolus at the Swallowing Threshold

Table 2 shows the number and percentage of persons able to prepare the food with suitable texture for swallowing and the means and SDs for the weight of a mouthful of food, mastication frequency, and mastication time for each food. The percentage for suitable preparation for swallowing was around 65% for boiled rice, boiled fish-paste, and rice cracker. However, two foods had low percentages for suitable preparation for swallowing: sliced white bread due to high adhesiveness and ginger-fried pork loin due to high hardness. One participant for boiled fish-paste and six participants for rice cracker did not masticate because of their fatigue.

**Table 2:** Food bolus at the swallowing threshold

	Suitable preparation for swallowing (*n*, %)^a^	Weight of a mouthful of food (g)	Mastication frequency (times)	Mastication time (s)
Boiled rice	21, 70%	10.4 ± 1.3	64.0 ± 28.6	42.5 ± 13.2
Sliced white bread	4, 13%	4.7 ± 0.7	59.0 ± 20.9	40.8 ± 13.4
Ginger-fried pork loin	7, 23%	3.8 ± 0.6	53.1 ± 20.0	34.8 ± 15.3
Boiled fish-paste	17, 59%^b^	7.7 ± 1.2	65.8 ± 29.1	36.5 ± 15.1
Rice cracker	16, 67%^b^	2.9 ± 1.8	51.1 ± 15.5	35.6 ± 12.0

Values are mean ± SD or *n*

^a^The suitable food bolus for swallowing was defined as texture that meets three criteria: under 15,000 N/m^2^ in hardness, under 1000 J/m^2^ in adhesiveness, and between 0.2 and 0.9 in cohesiveness

^b^One patient for boiled fish-paste and six patients for rice cracker did not masticate because of their fatigue

The associations between suitable preparation for swallowing each food and several variables, based on univariate and multivariate logistic regression analyses, are provided in Table 3. Suitable preparation of the food for swallowing was positively associated with the a* values in boiled rice, ginger-fried pork loin, boiled fish-paste, and rice cracker (Crude OR 1.18, 1.15, 1.17, and 1.50; *P* < 0.001, = 0.026, <0.001, and <0.001, respectively). The higher a* value not only meant that a person’s mastication was better but also that their preparation for swallowing was better. Age was not significantly associated with suitable swallowing preparation for any of the foods.

**Table 3:** Results of univariate and multivariate logistic regression analysis for the associations between suitable preparation for swallowing each food and several variables

Explanatory variables	Boiled rice			
	Crude odds ratios (95% confidence interval)	*P* value	Adjusted odds ratios (95% confidence interval)	*P* value
The a* values of the color-changeable chewing gum after 120 s of chewing	1.18 (1.07–1.36)	<0.001*	1.14 (1.02–1.32)	0.012*
Mastication frequency of each food	1.05 (1.01–1.12)	0.014*	1.03 (0.99–1.11)	0.195
Age (years)	0.97 (0.88–1.07)	0.587	0.99 (0.85–1.16)	0.893
Total score of the mann assessment of swallowing ability	1.00 (0.92–1.09)	0.906	Excluded	
Good occlusal contact status: Eichner index class A without removable good occlusal contact status: Eichner index class A without removable prostheses (No = reference)	6.00 (0.63–57.0)	0.204	Excluded	

Sliced white bread				Ginger-fried pork loin			
Crude odds ratios (95% confidence interval)	*P* value	Adjusted odds ratios (95% confidence interval)	*P* value	Crude odds ratios (95% confidence interval)	*P* value	Adjusted odds ratios (95% confidence interval)	*P* value
1.10 (0.97–1.43)	0.164	1.10 (0.97–1.43)	0.164	1.15 (1.01–1.45)	0.026	1.10 (0.98–1.39)	0.123
0.99 (0.93–1.04)	0.570	Excluded		1.03 (0.99–1.08)	0.168	Excluded	
1.05 (0.92–1.21)	0.461	0.95 (0.82–1.08)	0.462	1.05 (0.95–1.19)	0.317	0.97 (0.85–1.08)	0.534
1.07 (0.97–1.18)	0.187	Excluded		1.00 (0.90–1.09)	0.972	Excluded	
8.14 (0.72–91.9)	0.095	Excluded		9.00 (1.32–61.1)	0.026	4.79 (0.61–47.8)	0.137

Boiled fish-paste				Rice cracker			
Crude odds ratios (95% confidence interval)	*P* value	Adjusted odds ratios (95% confidence interval)	*P* value	Crude odds ratios (95% confidence interval)	*P* value	Adjusted odds ratios (95% confidence interval)	*P* value
1.17 (1.06–1.35)	<0.001	1.14(1.02–1.34)	0.023	1.50 (1.17–2.81)	<0.001	1.62 (1.18-4.02)	<0.001
1.04 (1.0006–1.09)	0.046	1.04 (0.99–1.11)	0.125	0.98 (0.85–1.08)	0.647	Excluded	
1.02 (0.93–1.12)	0.703	0.95 (0.81–1.13)	0.577	0.97 (0.87–1.07)	0.533	1.11 (0.82–1.66)	0.497
0.95 (0.86–1.03)	0.235	Excluded		0.92 (0.05–1.01)	0.082	Excluded	
9.78 (1.02–93.5)	0.043	4.07 (0.23–346.4)	0.359	5.44 (0.54–55.2)	0.189	Excluded	

Odds ratios of the a* values were per frequency

Outcome variable: suitable preparation for swallowing each food

After the univariate analyses, the a* values (main explanatory variable), age (spontaneous), and the significant variables were tested in a multivariate logistic regression analysis

### Cut-off a* Values of the Color-Changeable Chewing Gum after 120 s of Chewing

The cut-off a* values of each food, except sliced white bread, are given in Table 4. The specificities of the cut-off a* values were markedly high (1.0) for boiled rice and boiled fish-paste. Thus, their positive predictive values were indirectly high. The sensitivities of the cut-off a* values were high (0.86–0.94) for ginger-fried pork loin, boiled fish-paste, and rice cracker. Thus, their negative predictive values were indirectly high.

**Table 4:** Cut-off a* values of the color-changeable chewing gum after 120 s of chewing

	Cut-off a* values	Sensitivity	Specificity
Boiled rice	27.8	0.76	1.00
Ginger-fried pork loin	28.7	0.86	0.65
Boiled fish-paste	21.2	0.94	1.00
Rice cracker	21.2	0.88	0.75

## Discussion

Although many previous studies have evaluated mastication ability [6–14], the regular foods a person can prepare suitably for swallowing, based on mastication evaluation, remain unknown. This study shows that it can be clearly determined, via mastication evaluation using a color-changeable chewing gum, whether older adults can masticate and prepare regular foods suitably for swallowing. The high a* values were significantly associated with a high percentage of participants able to prepare food with suitable texture for swallowing for four of the five foods. For cut-off a* values, there were markedly high specificities (i.e., high positive predictive values) for boiled rice and boiled fish-paste and high sensitivities (i.e., high negative predictive values) for ginger-fried pork loin, boiled fish-paste, and rice cracker.

Thus, we believe that mastication evaluation using a color-changeable chewing gum is not only useful but also extremely practical, even for older adults. Mastication evaluation using a color-changeable chewing gum requires only a few minutes to achieve an objective evaluation and is used not only in laboratories and specialized institutions but also in a wider range of settings, such as an individual’s home. The gum color is more easily measured using a visual color scale than using a colorimeter [14].

Our results would be applicable to older adults with modified dependence in motion at home or in nursing facilities. We consider that food bolus texture at the swallowing threshold likely reflects real-life behavior and is more clinically relevant than a fixed mastication frequency. Occlusal status of the participants was consistent with an older adult population based on the Eichner index [21]. Reasons for admission of our inpatients were mainly rehabilitation after an acute phase of disease; thus, the general condition of our patients was stable. Activities of daily living for most of the participants were modified dependence in motion and modified independence in cognition.

The measurement of a food bolus has limitations; however, we consider these limitations to be minimal. There were missing values for the food bolus texture for two foods because several participants had low endurance. There is a possibility that the entire food bolus was not spit out and measured due to stage II transport [22, 23]. However, masticated solid food rarely enters the hypopharynx before swallow onset [24]. Moreover, if the food bolus texture in the oral cavity is suitable for swallowing, then that in the pharynx at the swallowing threshold would be suitable too [25].

Instrumental texture profile analysis is a good tool to assess textural parameters of semisolid foods [17]. If mastication is not suitable for swallowing preparation, then the food bolus would not be semisolid and cannot be measured by instrumental texture profile analysis. However, it was not a problem for this study because the main outcome variable here was the food bolus texture at the swallowing threshold, represented as suitable mastication for swallowing (binary variable).

For four of the five foods, the a* values of the color-changeable chewing gum after 120 s of chewing were significantly associated with suitable preparation of the food for swallowing. For sliced white bread, however, the a* values were not significantly associated with suitable preparation of the food for swallowing. Many participants’ texture of sliced white bread was not regarded as suitable for swallowing preparation because adhesion was too high (over 1000 J/m^2^), despite suitable hardness and cohesiveness. Adhesion might be changed by selecting different types of bread.

We regarded the texture that satisfies three criteria (in hardness, adhesiveness, and cohesiveness; see methods) as suitable for swallowing. These criteria have limitations. The criteria were not based on the results of scientific verification experiments; however, they were based on the observational studies that determine the texture-modified food pyramid according to the texture measurement of several levels of real texture modified food in a hospital in Japan.

In conclusion, this simple mastication evaluation would determine if persons can masticate and suitably prepare regular foods. This approach would lead to a reduction in unnecessary mechanically altered or pureed food for older adults who can eat pureed food and safely provide palatable food, which may have a positive impact on their quality of life.
